# Rhabdomyosarcome ou pseudotumeur inflammatoire

**DOI:** 10.11604/pamj.2014.19.75.4269

**Published:** 2014-09-24

**Authors:** Fatima Zohra El Meriague, Rajae Daoudi

**Affiliations:** 1 Université Mohammed V Souissi, Service d'Ophtalmologie A de l'Hôpital des Spécialités, Centre Hospitalier Universitaire, Rabat, Maroc

**Keywords:** Rhabdomyosarcome, tuméfaction inflammatoire, TDM orbito-cérebrale, Rhabdomyosarcoma, inflammatory tumor, Orbito-cerebral CT

## Image en medicine

Il s'agit d'un enfant de 4 ans, sans ATCD pathologique particulier, qui présente depuis 2 mois une tuméfaction inflammatoire, douloureuse, rapidement progressive de l’œil gauche. A l'examen, l'AV était à 2/10 et on retrouve une exophtalmie, axile, inflammatoire, irréductible et non pulsatile de cet œil. Une TDM orbito-cérebrale réalisée chez ce patient montre un processus orbitaire intra et extra conique au dépend des muscles droits externes et internes ayant un aspect fusiforme suggérant un rhabdomyosarcome. Devant ce tableau, une biopsie orbitaire a été effectuée en urgence. Elle a éliminé un rhabdomyosarcome et a révélé plutôt une pseudotumeur inflammatoire. Le patient a été mis sous corticothérapie avec une bonne évolution mais a gardé un ectropion qui a été ensuite pris en charge chirurgicalement. Le rhabdomyosarcome est le premier diagnostic à évoquer devant toute exophtalmie inflammatoire de l'enfant car le pronostic vital est mis en jeu.

**Figure 1:**
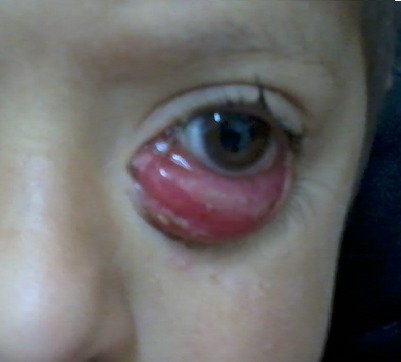
Exophtalmie dans le cadre d'une pseudotumeur inflammatoire

